# Putting Suicide Policy through the Wringer: Perspectives of Military Members Who Attempted to Kill Themselves

**DOI:** 10.3390/ijerph16214274

**Published:** 2019-11-04

**Authors:** Tirzah Parrish LeFeber, Bernadette Solorzano

**Affiliations:** 1 Veterans Health Affairs, Social and Behavioral Health, Anchorage, TX 99504, USA; 2 Psychology Department, Our Lady of the Lake University, San Antonio, TX 78207, USA

**Keywords:** military studies, policy, stigma, military suicide, military suicide prevention and policy, lived experience, qualitative, critical suicidology, psychology, military mental health

## Abstract

In response to the Air Force Surgeon General, Lieutenant General Mark Ediger’s call for medical services to be guided by service members’ values, preferences, and experiences within the medical system, we conducted an interpretive phenomenological analysis of transcripts in which service members shared their experiences of military mental health policy and practices after being identified as suicidal. Themes of their experiences underscore nuances as it relates to intersectionality of policy when faced with unique military contextual factors and power differentials; both of which were missing in available research literature. Their experiences also illuminate further the innate “Catch 22” which happens when accessing help. Catch 22 basically means if you know you need help than you are rational; but if you actually seek help, then you are crazy and not trustworthy to do your job. Themes presented center on the lack of confidentiality of Service Members in the Workplace, effects of Unit Members’ Surveillance and Command Directed Evaluations, and experiences of Military Mental Health Services. Critical discussions of policy and taken for granted assumptions that often drive narrow responses to suicide, treatment, prevention, and stigma are presented. Particular attention is given to the lived experiences of service members when placed under the demands of circumstances created by policy that may inadvertently lead in some cases to further suffering. The paper closes with recommendations from participants and the authors for policy makers and future directions in research.

## 1. Introduction

*Go ahead and throw money*

*You don’t hear me*

*You have all the answers, you see*

*But still you don’t hear me*

*You know it all booksmarts of society*

*Professionals are real good at talking, but not hearing me*

*As long as you can change my identity….*

*You label me with anxiety, depression, PTSD*

*This lifelong cycle by society is not capable of hearing me….*

*You’ll wonder why then you didn’t listen to me…*[1].

Hank Wentz

Suicide is viewed as caused by mental illness [2,3]. This understanding then creates a context for military policy and the way service members are treated to evolve from a hierarchical expert position of mental health researchers and mental health providers without input from the members seeking help [4,5,6,7,8]. The goal of our research was to take a different approach, agreeing with and responding to the Air Force Surgeon General’s call [9] that for continued transformation of military medical policy and services, it is vital and critical to ensure that the perspectives of those with experiences in our medical system are legitimized and considered in ongoing production of knowledge informing policy transformations. The perspectives of individuals formerly suicidal as applied to the creation of policy was spearheaded by several large civilian health organizations recently to good effect. Adopting the practice in 2016, the Air Force Surgeon General agreed and urged in a directive memorandum to medical leaders to create medical practices and polices responsive to the values, needs, and experiences of those we serve [9]. This perspective places participants in our military medical system in the role of key collaborators integral in the production of knowledge to inform policy transformation. This study included service members who experienced challenges created by the existing mental health policy and practices when they were identified as considering suicide. The exploration of their lived experiences is a critical means to broader based suicide prevention in military policy transformation efforts. Our work was completed to understand ways military mental health policy leaders can respond better to individuals and apply insider knowledges to the ongoing transformation of mental health policy and its practices, now lacking in their internal practices. To this end, secondary information—historical transcripts of individual interviews with service members who shared their experiences when identified as experiencing suicidality from prior research completed by Tirzah [7] were revisited for further analysis.

### 1.1. Evolution of Suicide Prevention Policy: Staking a Claim in Pathology

To illuminate why suicide has only been understood largely through a medical perspective in the United States, below is a brief summary of the historical evolution of suicide prevention policy efforts in the United States.

The first policy initiated for suicide prevention in the United States was created in 1999 by the leading medical authority, the United States Surgeon General. The United States Surgeon General’s call for action to end suicide helped cement the idea that suicide is a health condition requiring problem solving and action by mental health researchers and mental health providers. In response to the 1999 United States Surgeon General’s call for action, in 2001, mental health leaders in the U.S. Department of Health and Human Services, in collaboration with the Air Force Medical Service, and other health systems across the United States, published the first ever National Strategy for Suicide Prevention [4]. The report was updated in 2012 and continues the idea that suicide is a health problem that can be solved by improved surveillance of and identifying populations deemed at risk so that individuals who are perceived as mentally ill could be funneled to mental health services earlier. The 2012 national strategy will continue to guide the United States suicide prevention efforts for the next decade [6]. The Department of Defense 2015 Strategy for Suicide Prevention uses the framework outlined in the 2012 national strategy ([6], p. i.).

### 1.2. Implications of Suicide Staking a Claim in Pathology 

The assumption that suicide is evidence of a mental health disorder, or is an inherent marker of pathology for individuals considering suicide has fueled mental health research and treatment geared toward identifying demographic information and characteristics of people who commit suicide [10,11,12,13]; predictors of suicidality, and detection of suicidality [14,15,16,17,18,19,20]. While understanding suicide from the medical perspective has no doubt saved lives, it has inadvertently created a circular loop in the continued national and military policy responses of suicide as being largely understood in the context of mental health pathology controlled and privileged by mental health professionals requiring continued mental health problem solving [4,5,6,8], overlooking other perspectives, in this case service members’ perspectives of military mental health policy and suicide prevention efforts. Military leaders recognizing the lack of voices of those who have lived experience and are most able to inform what would be helpful in our internal military policy transformations, asked us to collect their experiences as an initial step to resolve this [7]. 

As individuals who practice narrative therapy, we agree with White’s view [21] that by switching from an internalized focus of the problem to an externalized focus, people may start to find different ways of addressing the effects of the problem in community. In this case, viewing service members’ insider knowledges that speak to the broader, external demands placed on them within the military context that are embedded in their experiences to inform our policy efforts. This research is our attempt in using our privilege as researchers, to stand in solidarity with those effected by the system and practices we are a part of, the research viewed as a critical platform for descriptions of lived experiences as a legitimate source to the ongoing transformation of military mental health policy and practices by inviting them to be a part of the conversation. 

## 2. Methods 

Our work was completed to identify other ways military mental health policy leaders can respond to individuals and apply insider knowledges to the ongoing transformation of mental health policy and its practices, lacking in their internal practices. The core of our research centered on the investigation of secondary information from existing transcripts capturing the collaboration with seven military service members who offered accounts of their lived experience centered upon what was helpful in moving toward wanting to choose life after being identified as suicidal in the military system [7]. The participants included seven individuals, three women and four men ranging in active duty service from two years to over 20 years. The occupational services varied as well as the military branch in which they served. Age ranges for those who reported were between early twenties and late forties. 

In this new endeavor, in an iterative process, using Interpretative Phenomenological Analysis (IPA) [22] the transcripts were revisited, pulling out descriptions of lived experiences specifically related to military practices from policy. The phenomenologists’ focus on a first-person perspective matches the research interests in legitimizing insider’s local knowledge in hopes to transform future policy. From a phenomenological perspective, service members’ worlds presented themselves in dialogue involving their stories, the transcripts—the mechanism in which this process was recorded—we revisited to further investigate the descriptions related to experiences with policy and practices. According to Smith et al., Interpretative Phenomenological Analysis (IPA) attempts to “capture particular experiences as experienced by particular people” ([22], p. 16). In other words, there is a focus on personal meaning-making within a particular context. Their experiences are legitimized, not because they must be experienced by a statistically significant majority, but because they are insiders to the experience of existing policy, what we want to hear about and learn from. IPA serves as the framework to approach service members’ unique experiences with military policy when identified as being suicidal. In a double hermeneutic interpretation, their experiences were understood as a response to the demands of the world entangled with the meanings that they attributed in which our understandings added to their meanings. In interpreting the stories, IPA enabled a strong commitment to understand the individual’s point of view, and we analyzed the transcripts by keeping closely to the language and perspectives provided. 

While IPA is mindful to obtain robust descriptions of lived experiences, it also aims to move to an interpretation of the experiences. In other words, IPA not only seeks to describe, but also to understand meanings and interpret the accounts gathered. In interpreting the stories by connecting them with military policy and practices, IPA held a strong commitment to understanding differing points of view. This was important so as not to treat their stories as data to be funneled and regurgitated through our professional language or privileged only through our methodical mechanisms. 

### Meaning Interpretations: Analysis

IPA emphasizes that the research exercise is a dynamic process with an active role for the researcher in that process. Access to individuals’ meanings depends on the researchers’ own conceptions to make sense of through interpretative activity [22]. IPA served to respect individual’s stories, considering their experiences as life stories versus research data. IPA processes allowed for the analysis of the transcripts to remain centered on the meanings captured in their stories without dismissing the fact that it did so in relation to our interpretations from our subsequent meaning making and interpreting their words in relation to military policy. 

In the current research analysis, drawing from IPA’s ideographic approach, an in-depth analysis of each of the participants’ stories within the context of military specific mental health policy and practices was completed. We followed the below steps, congruent with IPA processes outlined by Smith et al. [22]:

(1) Each transcript was read individually twice with a focused interest of pulling out the descriptions and experiences related to the military workplace and services, with emergent themes created in the margins for each transcript
(2) Placed together in an excel, one tab for each person, all the experiences related to policy were captured with emergent themes with beginning dialogue of what is common and not so common among the group
(3) The development of moving from individual analysis to group analysis, placing themes and looking across the group, connecting experiences to policy mandates.
(4) Organization of a paper trail, from initial review and comments of transcripts, emergent themes, and quotes, tracing from individual to group analysis
(5) Development of full narratives, drawing heavily on participant descriptions

Tirzah completed the themes, with Bernadette’s guidance along the way borrowing from Morrow and Smith’s processes of rigor [23]. Tirzah included mechanisms such as the creation of audit trails for categories of meanings, and creation of themes for Bernadette to follow. We are also visible in our writing to allow, as much as possible, the perspectives of the service members, alerting the reader to form their own judgments. This work does not claim knowledge authority and we realize that other readers may, based on their lived experiences and perspectives, find new fantastic things to explore or to bring forth new knowledge from engaging in reading this work. 

## 3. Collaborators’ Lived Experiences Categorized into Themes 

Within the themes (Table 1) are descriptions of service members in bold that attracted our attention, thereby drawing your attention, being visible in our focus of creation of themes and subsequent analysis through this writing. 

Embedded in the themes we underscore the policy, congruent with the aim of the research endeavor, for policy makers to understand insider experiences of policy to transform future policy.

### 3.1. Theme One: Lack of Confidentiality of Service Members in the Workplace

One of the themes described is related to the loss of confidentiality in the workplace. There are several differences in military policy compared to civilian policy related to confidentiality. In accordance with the mandates outlined in Department of Defense Issuance (DODI) 6490.08, military health providers must disclose, under specified notification standards, health information to service members’ commanding officers/supervisors and include “the diagnosis, a description of the treatment prescribed or planned, impact on duty or mission, recommended duty restrictions, the prognosis, any applicable duty limitations, and implications for the safety of self or others” [24] (p. 6). The experiences described below are circumstances created under mechanisms outlined in Department of Defense Issuance 6490.08. 

#### 3.1.1. Subordinate Theme A: Ripples of I’m Tired of Being Told I Can’t Do Something When I’m More than Capable of Doing It

**Trish:** *So, I was kind of labeled, like when you’re in high school and you’re labeled as the kid that can’t do anything, put in the corner. That was me. Everybody treated me like I was some fragile victim that they couldn’t trust to do anything because they didn’t know when I was going to fall apart. When I had proven to everyone that I didn’t have panic attacks at work, **I was still fully capable of doing my job and I fought for three years to prove to everyone that I was capable of living and working and it was so stressful and so time consuming and drained me so much all the time** because **nobody wanted to trust me or give me the big project that would help me with my career and they wanted to give me the office filing work** because they didn’t want to put me under too much stress…so **when I got to my second base I didn’t say anything** about my past”.*

**Gummo:** *To take it even further as a part of pararescue men, **if we go and seek help, they ground us so now we cannot do our job**. If I go and tell them that I have nightmares of combat experiences, **they take me off the flying status and I cannot do my para-rescue duties so it further isolates and reduces meaningful existence**.”*

Hoppi shares how being taken off the team leads to not exposing one’s suffering:

*Most important is on a team, a Special Forces team, a Green Beret team, whatever. Any form of mental weakness, especially, when I was serving in these rotations and stuff, number one, your security clearance was at risk and folks still even feel that way, even though that taboo is supposed to be gone**…if you take them off of that team, you just took their life. What they’re going to do is self-medicate. They don’t want to expose that to anybody, to their wife, anybody**.*

In Hoppi’s experience, hiding included: Dr.:

*As a matter of fact, when I went to the ER…and I was having all of these physical things going on, one of the Flight Surgeon doctors called me and he said, **“I’m going to give you a prescription of this stuff called “planipan” or whatever it was called and I’m not going to put it in your medical record…***

Aria also described people wanting to hide so they can be perceived as normal:

*…But I do understand why people don’t seek help. Especially military soldiers, because I’ve seen sometimes people treat the military as their sole career and it is probably their last option or choice and they don’t want to screw it up. **The military is all about perception, and they don’t want anything that is deemed dysfunctional or defective…Anything that’s not considered “normal” is dysfunctional or defective, and that is not allowable in the military. Some people try to hide it.***

**John:***My sister is a psychiatrist, so we would joke about it for years. “As soon as I’m done with the Air Force, my first appointment is going to be with a psychiatrist so I can get on pills.” We would laugh about it, but that’s really how I felt. **I’m a pilot, so it’s against our nature completely to accept medication of any kind because that involves waivers, you’ll be grounded**…There’s a negative stigma with it. I wish it wasn’t that way, but if you’ve got somebody who says…It’s common sense that the squadron is full of type-A personalities, a bunch of pilots. If a guy says, “I’m a depressed case. I see a psychiatrist. I’m on these pills. I can’t fly for six months until I get a flight waiver approved,” it’s like, “Oh, jeez, dude. Sorry. **Go sit in that back office over there and work on awards and decorations until you get your life figured out and can fly again.”*** (imagine writing awards and decorations for other members in the unit to progress in their careers, as your career is diminished). *That’s the harsh stigma of being in.*

Trish describes how viewing what happens negatively to others undergoing mental health services sustains the stigma:

*The stigma is service wide. I think it’s less than it used to be because there has been a lot of success with it but at the same time when **good people are put through the wringer for seeking the treatment that just increases the stigma and it makes people want to not recommend it and not do it themselves because it can have a huge negative impact as opposed to just figuring things out on a different level**…If I would have known that this whole situation would have happened there’s no way I would have went to mental health and **there’s no way I would have recommended it to anybody else either**. The stigma is use.*

Gummo and Hoppi describe how stigma may change when considering the intersections of age, family, warrior ethos, and phase of career contexts:

**Gummo:** ***Just a young infantryman, a young grunt, he wouldn’t want to speak up because then he thinks his weak**. Whereas most of the guys **in my career they’re a little more mature and so they see past that. It’s not weakness, it’s just really just basics of, “Well, I’m ending my career if I do this.**” It’s not who gives a shit if someone thinks I’m weak or not, that’s irrelative, it’s career ending…**When they’ve got a wife and kid that’s hard to swallow.***

Hoppi also underscores military contextual features that influence the performance of hiding from others when considering the intersections of age, the Alpha male ethos, and power differential inherent to his higher rank:

*…If you think that I’m embarrassed (for taking psychiatric medication), you’re out of your mind, but it was a taboo and a stigma. **At that point in the game (nearing retirement and higher ranking), I didn’t care because I figured, who’s going to come up and challenge me? Bring it, is the way I looked at it**…I do yoga. **Would I come out here and tell a bunch of Green Berets on my team that I go home each night and I’m doing 30 minutes of yoga? No. I might now, but back in the day? Absolutely not.** You went to the gym, and you ran 20 miles with 100 pounds on your back and threw it off and said, “That didn’t hurt.” You’re not going to say, “I go and watch the sunset.” No. It’s just not something you do. **You’re surrounded by warriors, and at a minimum, you’re going to maintain that outward appearance and that dominant, Alpha male presence, period**.*

McKenzie highlights that military messaging to dispel stigma may inadvertently create it due to actions not matching the messaging. She also highlights the nuances created with the idea of being honest.

*I would love to see that change because you can’t persist and say, “Hey we’re here to help you, we care about you, you’re a member of our family” if you will but then turn around and completely disregard everything you just said. That’s my two huge things is that as long as we’re still able to do what we’re supposed to be doing we shouldn’t be judged any differently. **The fact that the whole Air Force philosophy of being honest and open and seeking help and being a wingman that needs to be followed. It’s not one of those things that you can just say because it sounds good. That’s why there’s a stigma is because people are concerned that they’re going to be just treated differently, that they’re going to risk their careers.***

#### 3.1.2. Subordinate Theme B: Units Being Super Supportive and/or Not

Another subordinate theme related to loss of confidentiality in the workplace in accordance with Department of Defense Issuance (DODI) 6490.08 [24], illuminates the impact of the service member beings supported and/or not in their units after their unit is notified, they are considering suicide. Trish discerns how she is treated differently in her old unit being a victim in one and not in the other:

*…**I had to tell them what was going on** and that’s when they were like, “You could have told us from the beginning. **Everything’s okay. We’re here for you. Here’s our numbers. Here’s this. Here’s that. Hopefully everything works out**. You should check in with us while you’re gone.” And **that’s when I realized that I could have told them the whole time, but because of how terrible the experience was at my first base** and how I hated feeling like a victim, **I didn’t say anything**…*

Trish discusses how a supportive work environment and honoring her privacy is an important contextual feature in lowering stress and anxiety:

*…**They also valued my privacy which was a big thing because in the military you really don’t get a lot of privacy.** At my second base the leadership was really big on making sure that even when they called me into the office to talk about some stuff, they would say that they were going to talk to me about something else just in case somebody heard something. **They’d be like, “Hey, do you have a minute to talk about this thing that we’re working on?” And I was like, “Yeah. Sure.” Go to their office and they’d be like, “Hey, are you okay?…That cut down on a lot of my stress and anxiety because I was able to actually trust some** of the people that I worked with which, **at my first base I wasn’t able to do**.*

*….**It was the best people ever. I felt a lot more support. I wasn’t as depressed all the time because I wasn’t just dreading going into work because I enjoyed going to work. The people cared; the people were nice. They wanted to help, and they knew that I was going through a lot and they weren’t treating me like I was different. They were still trying to give me a job to do** and keep me busy and do as much as they could on the side to make sure that I was okay.*

McKenzie highlights below that the loss of confidentiality in her workplace led to being isolated, losing social support, and being treated differently in the unit. These risk factors are known to place individuals further at risk for possible issues with mental and/or physical health [25,26].

**McKenzie:** *Things were at their worst; my command went from super supportive to non-existent and my first shirt (sergeant) and the other co-worker have been really the only two who’ve not abandoned me in the whole process….Take for instance the first time I was in-patient my command visited every week, like older colonels visiting driving two hours each way. They visit every week; they called every week and **then the second time not only did the calls never come the visits only came when they had to. When I got out they literally isolated me from every person I knew.** They themselves would sit and look at me with one of those, yeah, okay I’m hearing it or I’m listening but not really paying attention to what you’re saying type stares. There was never two-way conversations. The fact that even today one person from my old unit we talk, there’s a couple of people that will be cordial and they’ll be like, hey how was your day kind of thing but never in-depth about anything.*

***That’s the majority of the people won’t have anything to do with me. They literally will walk down the hall right past me not smile, not nod, not say hey, how was your day? You treat strangers better than my old unit treats me and I never did anything wrong. That whole we’re here for you line is a complete load.** I don’t know, that’s huge for me because I was just like I know at least in the past being in the service it meant something. **It was that family, that sense of security and pride and it’s turned a huge corner** and becoming just another corporation and yet I still want to stay.”*

*…Okay, **we’re going to get you through this.” Then to turn around and have them completely abandon you it’s like, oh, another learning curve of just because somebody starts out showing that they care for a lack of a better word doesn’t necessarily mean that they’re true and loyal** to what they’re saying. Their actions have to continuously match their words.*

John describes his different experience in the context of power differentials when working directly for the commander and thereby getting a slap on the wrist:

*….It wasn’t as negative as I thought it was going to be. I was expecting I was going to get out of that detox ward, I’d have letters of reprimand, article 15s for dereliction of duty, all of this stuff. I was very paranoid that the bender I went on…I called off sick for two days while I was going through this bender. I was paranoid about that. **It was a really different response from my chain of command. I’m an executive officer at a group level, so I’m working directly for an 06 (Colonel). He came down and saw me. His response was pretty amazing. He didn’t give me any formal paperwork. There was no letters of reprimand, article 15. He basically gave me a slap on the wrist.** About the most negative thing that happened was, they had me in line to be a Director of Operations at one of the XX squadrons, so he put the kibosh on that.*

*…It was a real good wing-man concept. In a way, it was a success story for the leadership. I felt that’s why they praised it. Here’s a guy who’s struggling. Here’s somebody who intervened. Self-admitted, got treatment. **They said, “Given the circumstances, you did the right thing. If you do it again, you’re going to get crushed.”** That’s basically what they said.*

We are curious who is getting crushed the first time, not having the power and privilege that John carried.

### 3.2. Theme Two: Unit Members Surveillance and Command Directed Evaluations

Another theme centered upon the culture of surveillance within the workplace and being placed under demands to undergo a mental health evaluation by those who have authority over the service member. In the military, supervisors and commanders in the workplace have the authority to order subordinate service members to be mentally assessed “for a variety of concerns, including fitness for duty, occupational requirements, safety issues, significant changes in performance, or behavior changes that may be attributable to possible mental status changes” [27] (para c, p. 2). In addition, in accordance with DoDI 6490.04, all service members are educated through annual training “regarding the recognition of personnel who may require Mental Health Evaluations for imminent dangerousness, based on the individual’s behavior or apparent mental state” [27] (para g., p. 3). 

Aria underscores the difference between an old commander and a new commander’s actions. She also describes how the command-directed evaluations can be initiated (or not) as a means of social control for desired behaviors. This suggests that psychological or psychiatric diagnoses and treatments can be used as punishment from higher ranking service members as a means of maintaining social control over subordinates:

*My previous commander gave me a set of initiating protocol, like the **Command Directed Evaluation (CDE), I had to get a CDE evaluation where the mental health clinic was basically in charge of stating if I’m still able to continue my career**…he did initiate it. **He saw that I was seeking treatment and he saw improvement. He didn’t think it was necessary (to be discharged). I got a bunch of awards, I passed my test**…what initiated the (2^nd^) CDE evaluation for the briefing I got from my new commander, is he saw that I was receiving…**The (new commander) saw that I was getting, not written up, but counseled a lot, like verbal counseling from my supervisor over minor, trivial stuff like hair…**what else…and there was other things that were mentioned that I’m fighting against because the way she (supervisor) worded it made it seem like it was just over exaggerated and false information. He was wondering if all those allegations that were made by supervisor was regarding my mental state. I’m like, “How is having your hair wrong…” and these allegations, that I propose are wrong, contribute to my mental state? I’m perfectly fine. You know?”*

Trish shared the tension she has experienced with power differentials and how her low rank situated suggestions from those higher ranking as a threat that if she didn’t heed the suggestion, negative outcomes may have followed:

*The day that I went to see mental health had been a really tough week for me and then my supervisor, flight commander and flight chief ganged up on me unexpectedly and were like, “You need to see somebody either the chaplain or mental health”…not forced me to go but basically said, “Hey we’re telling you to go kind of thing.” Once I was there, I’m like, ‘I might as well just be honest and say everything that’s been going on kind of thing.’ **I think it was to the point where if I didn’t volunteer to go they would have command direct me to go so again that’s one thing that I don’t want any kind of negative outcomes because they’re telling me I have to go. If I volunteer then that shows that I am straight up and willing to get help and just being the honest, straightforward person that we’re supposed to be as we serve.***

Aria, in the same vein, when confronted with gossip about her and others, was educated that standing up for herself to her peers meant they could raise alarms about her to her leadership.

*“They also got involved…what really set it off is **when I had an altercation with these two airmen that I caught gossiping at work about myself and my…it was not only about myself, but it was also about the Non-Commissioned Officers (supervisors) during that time. I caught these two airmen gossiping, I got upset and I confronted them…and there was a portion that was mentioned during their little gossiping session about why I was going to so many appointments,** because I had a high interest list. I told them why I was on the high interest list.*

*I told them the reason I was always going about, running about, was because I guess that I could trust them, like I could involve that information because I could trust them. I told them the reason why I’m gone so much and the reason why leadership…although they know about it, it’s okay with them, you don’t have a right to be doing this because you really don’t know what all is going on. The reason I’m doing this is because I’m on a high interest list which is for people that are on suicidal watch because they are thinking of attempting suicide or have suicidal thoughts and I guess what rung into his mind was, “Oh, suicide, suicide, suicide,” you know?”*

***He set the alarm for everybody. He contacted my first sergeant, he contacted my supervisor, both of them did. They didn’t call my phone because I was so tired of arguing with them and teaching them what is right and what’s wrong, that I just left because I was exhausted. You know how you hard it is to educate, I was like you know what, I feel like I’m just repeating myself, I need to leave and just take a break, so I just left. I was going to somebody’s house, a friend of mine…another airman’s house that I was okay to talk with at that time, because there’s usually a person who I would go and vent to and I was on my way, I get phone calls from not only my supervisor, my first sergeant and stuff like that. “Are you okay? You’re not thinking about committing suicide, because that’s what the airman told me, that you were thinking about something…” you know. I never said I was committing suicide; I’m just going to an airman’s house right now.***

Hoppi shared his experience as unit leader, using his power to educate others on ways to help without exposing the service member and exacerbating the situation:

*At that time, it was an education piece for me, as the leader of the company, was I brought them all in there I said, “It pains me to be on the lookout for this. Yeah, there’s nothing wrong with drinking a beer after work or whatever, that is still a normal thing, but when you see a guy walking around with a cup all day…You need to realize that we have been at war for 15 years and maybe he’s doing exactly that. Maybe he is self-medicating. **I’m not saying send him to the shrink because he’s not going to go. He just won’t drink in front of you anymore, he’ll go hide it. You can be a little bit smarter and try and out shrink the shrink there a little bit…The leadership in these environments need to me more in tune with their people, and when they see these red flags, figure out methods to extract information and even provide assistance without letting on that you know what the problem might be because that would mean that he’s exposed now and you could even exacerbate the situation.***

Hoppi further explains why, in his units’ unique contexts, surveillance of members for possible issues and if suspected, being told to report for a mental health evaluation would not work in his unit:

***That wouldn’t work here. It wouldn’t work at all because that individual, again, his intelligence level is way up there or he wouldn’t be here. He will go over there to check that block, he will convince the shrink that he is beyond good,** and he will come out of there and it will cause more damage. I’m not trying to say these are a bunch of Einsteins around here, but we purposely select above average intelligence folks, and the guys that are here are extremely intelligent. A lot of them have advanced degrees, masters, doctorate degrees, and they’re E6s. You’ve got some crazy smart people here that know how to…in special forces, probably one-fifth of the job is your ability to bullshit somebody. That’s just a fact. You’ve got to go work with indigenous people from other countries and convince them to work for you or die for you to make their country better. If you put that into context, it’s pretty easy to navigate around a psychiatrist.*

Unique to *Hoppi’s* experience was the intersectionality [28] of his leadership role, his unit’s warrior ethos, and how these factors played out in the interaction between him and his commander:

*When I came back from the ER, **the whole command knew it was all stress-related, and two weeks later, I was back fighting in Afghanistan. Of course, I would’ve have wanted it any other way, but somebody in that command should’ve said, “You’re going to take a break.” They should have. I would have fought them tooth and nail, but the reality is they should have said, “There’s red flags going off all over this guy, now to where it’s translating into physical problems. He’s thinking he’s having a heart attack and we’re just sitting there yapping,” I was sitting there talking to my company commander. Next thing you know, I’m back charging up mountains two weeks later. He didn’t know no better, and of course, he asked me, “Are you good?” What do you think I’m going to say? “Absolutely, I’m golden.”***

### 3.3. Theme Three: Military Mental Health Services: Diagnosis and De-Contextualization of Experiences

#### 3.3.1. Subordinate Theme A: Loss of Ability to Articulate Humanity to the Authority of Pathology Language

Another theme described the experiences of being limited to the words to describe life through the Diagnostic and Statistical Manual of Mental Disorders (DSM-5). That is, having their lives reduced to only words pathologically focused, funneled through the disordered dictionary used by mental health providers to language lived experiences of those that are mandated to seek services due to the idea they are mentally ill for considering suicide. In the military, if you are identified as being suicidal, you must report for mental health services for fitness of duty considerations.

McKenzie:

***The thing is with the diagnosis it’s…because I’ve read everything. It’s not so much that I have the disorder; it’s the way that they worded it that it’s so severe that I can’t function in a military environment which is completely BS (bullshit) because I am doing just fine. It’s the fact that here, here’s this diagnosis and we’re just going to lop all kinds of crap onto it to make it seem like you’re completely unstable…***

*Then the paperwork dropped for the discharge and I filed a congressional…and I filed a congressional complaint. After that, she (the military mental health provider) was like, ‘We can’t work together anymore’ because it was like me attacking her license and decisions, which is the exact same thing they were doing to me…*

***As I said earlier, the first time I went to mental health the two providers were anything but therapeutic or sympathetic or I felt very attacked by them just based on what they were saying and how they were saying it. It made me shut down where I’m like, “I’m not going to say anything”.***

Aria:

***They diagnosed me as…well, they diagnosed me on base as schizotypal disorder, a schizotypal personality disorder, where I…very odd, had an odd…which is very…I just don’t agree with…Well, there’s a diagnosis that you really don’t have proof that you had that diagnosis. I got a different diagnosis from taking a test, two tests from the VA hospital, and I got a diagnosis, and it said those tests were valid. Therefore, this is what she has, we constructed a treatment plan to help her, but when you share those same notes to somebody else and they come up with a different diagnosis and they try to construct a…**I guess their treatment plan, (on base mental health) they called me schizo typo and just don’t agree with it. **I don’t agree with what you’re saying**. I don’t agree with both of them because I’m neither nor one of those things. I’m neither avoidance or schizo typo, because later on in my visits, in my follow up visits with my VA clinic for the VA psychologist at the hospital. He filed my improvement because they had me on personal treatment at another clinic, and I told the lady that I just don’t**…I stopped taking treatment at that time (on base)…***

Aria describes the assessment process she was ordered to undergo by a new commander and supervisor after learning she was considering suicide and seeking mental health services:

***When it was reported to the new commander…the new commander ordered a Command Directed Evaluation (CDE) on me, so he scheduled…he called me into his office, briefed me on what he was about to do, the very next day I went up to the mental health clinic where my supervisor…because I had new supervisor at that time too…he got me to the clinic,** they had me sit there and do a 560 question test. I don’t see how it would normally take 45 minutes to do a 560 question unless they were really bored, and they just started clicking. It took me at least two hours to complete…**When I was done a psychologist that was there, I’d never seen her before…I never talked to her, I never had any visitation, she was this person I’d never seen. She asked me some questions I guess to get a sense of my background, then she dismissed me for lunch and I came back and heard the results of the test and she told me that it seemed like I was making over…the answers I was giving or putting on the test, like I was over esteeming myself, like over…shining myself in a good light. It was based on how I was feeling that day. I was going based on how I was feeling. There’s a big difference…She said, ‘it was just how the test was…’ in the test, if you answer questions that normally other people would have answered the same way, it will trigger something within the test to make it seem like you’re lying or you’re trying to go with the test…I was upset because I was just thinking about going through all of the questions all from that day, I was so upset. Of course, they probably documented my response to that. I was just so upset. I think anybody would be upset when she put it on paper, it made it seem like I was just…I just went off, or something like that. That I was unstable.** She was going to use my previous test I’d taken at the VA clinic, and **I pushed…why would you use answers from my test, you know, use answers so that I take another test? Why can’t they just use the one on how I’m feeling now?…I’m like, “It’s not about the test, it’s about my improvement…improved over the month. You’re not looking at my improvement, you’re looking at when I was not feeling at my best.***

Aria’s words speak to assessments locating the service member as being forever static, stuck in time in their worst moments, and having it used in present moment as ammunition that she is lying for improving from before. It is also another example of the tunnel vision that can happen when a diagnosis has been given and no one is taking into consideration the person and not the label.

***Gummo:** You’re a liability to them and that’s really what the function that they serve as. A provider for the military is basically just there to judge liability or not…*

*…**Yeah it’s difficult and then at the end of all that, at the end of your career you’re like, “Okay here’s what going on.” (turning in all the civilian healthcare documents). They’re turning in all these papers. They are going to turn in all of my documentation. It’s here you go. That’s very telling. It’s like, “Holy smokes” and that’s the position that I’m in right now.” You’re like, “Okay, you’re going to get medically boarded (medical panel that makes decisions on fitness of service member to continue to serve).” It’s like, “All right, do whatever you got to do.”***

#### 3.3.2. Subordinate Theme B: I Am Your Textbook and Theory, Study Me

Another theme centered upon hearing the same theory from multiple providers and group sessions and consequently services were not being helpful. The VA/DoD Clinical Practice Guideline for Assessment and Management of Patients at Risk for Suicide recommend only treatment options that fall under the Cognitive Behavioral therapy conceptualization using a structured framework, or medication [8]. The VA/DoD Clinical Practice Guideline for Assessment and Management of Patients at Risk for Suicide was updated and released in 2019, after the interviews were completed. It proposes the continuation of utilizing only CBT conceptualization and interventions in psychotherapy for individuals with self-directed suicidal behaviors.

*Charles:*

*…All textbook, it’s all, you go to school, and it’s all textbook. It’s an ongoing training for doctors and psychiatrists, and psychologists, but the true professional can get into my head. The true professionals can get in there, and help me, just Google in the word help is, it’s not going to be there. **It’s not going to work. The textbook…When you sit down with different doctors, and different psychiatrist and different psychologist. Hearing the same thing, over and over and over**. Finally, you’re sitting back, and you’re kind of laughing about it, because nothing has changed. Everything is textbook, and it’s a check of luck and move on. Now I understand that this conversation that we’re having right here, is to maybe push this mental health, in a different direction, I think it needs to be pushed in this different direction.*

***…It goes back to the getting of our conversation; they could research job or the individual prior to trying to help them**. Go watch. I challenge you ladies, go watch History Channel, or Google, we go back to that Google, we can Google anything. Special forces, green berets, rangers, Navy SEALS, watch their training. **Get a perspective…I think some of the things that they (mental health providers) would be missing, (my) life experience. Go see, go see…Have them therapist go see the training. Have these therapists go out of their office, go to the location of soldiers, get a little bit more perspective of what they do day to day, their mentality…***

Hoppi:

*I would say this, **and I’ve told this to several mental health providers that I’ve dealt with as a Sergeant Major. If you truly want to help these guys, especially where I’m at… Actually, it doesn’t matter where the person’s at, you need to look at the organizations that they come from and try and do your homework and understand the organization and what they do. Because my biggest frustration point is sitting there talking to somebody, and they have no idea in what capacity we function and what we experience and how we live and breathe.***

*I would say do the homework on the patient, and in the military case of this, the organization in which they come from. **Because each organization (and person) is different, and some of them are extremely intense like, the one that I’m sitting at right now, and others are very passive or pleasant or whatever. I’d say research the organization, what they’ve done and how they function, because you might better understand the person.***

***…Because you feel like you’re not wasting your time. Like I told the military family life people is, if I’m going to take two hours out of my schedule, which I don’t have, to come to your office and talk to you, and you’re just going to tell me two or three things that came out of a book or I can Google search, what is either one of us getting out of this? Nothing.** If you can understand me and get me to open up to you a little bit and dialogue with me and speak to me on the same level, for example, it’s a little bit different.*

*In my personal opinion, a psychologist or psychiatrist should be an extremely intelligent person, and if they are, which I’m sure they are, they should do that homework and figure out, ‘How do I get into this guy’s brain a little bit to get him into the box that I want him at so I can help him?’*

Gummo describes how he was trying to articulate his experiences in session, and the wrong fit led to him desperation and consequently, considering suicide.

*Again, I felt like talking to the therapist despite their good intentions and their dedication to the profession, I truly felt that it was fruitless…I immediately felt like I was barking up the wrong tree because whenever I went to see a counselor and not to minimize this person’s qualification or their experience or who they are as a human being but it was a really bad fit…**I was trying to articulate some of my combat experiences to her and the surreal nature of my nightmares and my grief issues and I intermingled this with the overwhelming situation I have with having a special needs son. It was just a horrible fit, in fact I walked out of the first appointment. I was really disenchanted with it.***

***I think a lot of my suicidal ideation came directly from the desperation of not seeking adequate help but the people that…What I mean by that is me not seeking but whenever I would go to the providers, they would completely offered no help whatsoever.***

*…I wanted to feel as if someone understood what I had gone through and what I was going through and I did not feel that that was adequate and a lot of the providers themselves they were like ‘Holy smokes, this is surreal.’ In a very real way for us to even hear what you’re saying that you’ve been through let alone us attempt to help guide you through your emotions on this.*

*…Again, it’s all about finding that right fit obviously but it’s very hard when no one else has experienced the things that you’ve experienced and so now they’re just applying theory to you.*

McKenzie describes how being vulnerable and sharing information to the military mental health provider limits her ability to articulate due to the lack of confidentiality and its effects:

*…**I need to feel that safety to know that I can talk about what’s going on and something positive is going to happen instead of either not talking or talking and it being used completely against me**…It’s just them doing their job, which is very difficult when you’re in a very vulnerable place.*

Aria found the services helpful but stopped treatment because she heard it before:

***… I stopped taking treatment at that time. I didn’t want to do treatment anymore because it was almost as though she was, like I heard it before from the group therapy session**.*

## 4. Researcher’s Conclusions and Responses to Participant’s Descriptions 

The insider knowledges were extremely useful to identify issues we had no idea were occurring in mandates from policy and clinical practice guidelines. What we appreciated about the descriptions from the stories of the service members were the nuances they highlight of the performances of policy. How policy played out was different in their unique contexts. Performance of policy was effected when entangled with power differentials prevalent in the makeup of the military ranking system, effected by the role they and others occupy, effected by the mission of their unit, where they are in their career (i.e., nearing retirement), developmental stages of life and differences in age; and what characteristics and social norms are preferred in the military (i.e., strength, honesty, integrity, and the warrior ethos) [29,30,31]. Such rich descriptions of insiders’ experiences within military policy and mental health services is missing in the available research and internal military policy transformation mechanisms. 

Their experiences also illuminate further the innate “Catch 22” which happens when accessing help. Catch 22 basically means if you know you need help than you are rational; but if you actually seek help, then you are crazy and not trustworthy to do your job [32]. This located the service members as unstable, with policy that also mandates them viewed and treated as such through placing them in menial job roles, unable to perform their mission or job duties, negatively effecting relationships with peers and others in the unit, and fear of being discharged and the ripples it would have to their families. There are also descriptions of abuses of power related to the command directed evaluation and allowing for supervisors and others of higher rank to direct subordinate members to present for mental health services or psychological assessment based on perceived wrong doings, which situates psychological assessment and services as a form of punishment. The descriptions of service members highlight military unique sociocultural features arising from DoD policy that contribute to the creation of stigma distinctive from that described and understood outside military contexts.

### 4.1. Criticality of the Language of Suicide with Pathology and Narrow Specificity of How to Help

The participants underscore unhelpful experiences when providers designate life problems only understood through the framework of mental disorders, disordered thinking, and narrowly locating issues of suicide living within the skull of the individual. They describe no curiosity of their life contexts nor looking outward to the social practices that create human suffering [33,34]. This idea contributes to the ongoing restrictive perspective of suicide and its prevention, to include stigma as being due to irrational thoughts and behaviors from disordered service members, thereby solutions are provided accordingly, (i.e., people experience suicide because they have undiagnosed mental health disorders, identify them so they can go to treatment, ending the problem of suicide). While the dominant medical perspective and pathological conceptualization of suicide have no doubt saved lives, it is apparent there are ramifications and inadvertent ripples to the lives of service members when being mandated by policy to be understood in these perspectives only. 

The service members’ descriptions speak to being dehumanized, with lack of control and power over who to share their stories of suffering with due to unique military specific requirements that mandate those identified as experiencing suicidality to be exposed to leaders in the workplace. They describe losing authorship to their stories to the authority of mental health providers, commanders and supervisors, who often through their practices, deem suicidality as evidence of a disorder, locating the issue inherent to the pathology of the individual. The service members storytelling is silenced, not legitimate, dead from an act of storycide [35] killed by the mental illness story.

The service members also speak to the same theory and textbook like mental health services they are mandated to use to help, as unhelpful. In the VA/DoD Clinical Practice Guideline for Assessment and Management of Patients at Risk for Suicide, CBT therapies, which are often manualized and scripted, is suggested for providers to use with the intention to “Reduce current unwarranted practice variation and provide facilities with a structured framework to help improve patient outcomes (prevent suicide and other forms of suicidal self-directed violent behavior” ([8], p. 2). What this language does is locate the person’s unique articulation in their own preferences to describe ways of living in their own contexts as not fitting in with the session’s “structured framework”. Consequently, the session is not helpful to the individual, seemingly helpful instead for the researcher interested in standardization of processes, helpful to mental health provider’s mandated by clinical guidelines to conceptualize problems in the CBT framework coupled with disordered language to understand the person’s suicidality. This seemingly creates services, and processes thereby tone deaf to learn the many changes and nuances of power differentials and intersectionality of unique sociocultural contexts of suffering illuminated in the service members stories. The service members are not allowed to articulate their experiences but are rather placed under the linguistic demands of how to speak about their lives through our own conceptualizations, “structured frameworks”, and DSM-5 disorders. When you look at it from the perspective of our participants it becomes an issue of social justice because they are being reduced to their labels, told what they need to do to get better, “replace thoughts with more realistic and useful ones” while ignoring the outward causes of suffering in addition their goodness of fit for the process. Reynolds illustrated how medicalized language “seduces us to abdicate our social and collective obligations to change the contexts in which these kinds of deaths occur—violence, poverty, (military unique social experiences) and homelessness, none of which are natural. This can also direct all research and resources towards a corporate medicalized response to what are primarily social issues” [36] (p. 176). 

In response, we share the service members’ own stories, in their words and life theories of understanding their consideration of suicide. This is what seems to have been lacking in their experiences in military mental health services: 

**Aria:** Aria shared that she joined the military looking for help “because I didn’t have any health insurance or medical benefits, and I couldn’t afford it”. She described her role in the Air Force as “customer service”. She underscored her religious belief as a contextual feature to “when it comes to suicide, with God and suicide, it’s because you get angry at your creator sometimes. Something…you know, life throws you lemons, and just get angry at your creator, and it’s so unfair…It’s like, you want control in your life, and you hate being not in control. You get angry.” 

**Trish:** Trish shared that she had served the prior six years in the Air Force in the medical field. She shared contextual features that contributed to her considering suicide: “My family is like very, very big on military history. My grandad was in, my dad was in and now I was in and I had been sexually assaulted and raped four times (by men with authority in the military) in a three-year period and by that time I had contemplated suicide…I was in the middle of the situation and I had told my mom about what was going on. She didn’t know about everything, but she knew about parts of things and she was disappointed that I had turned one of the guys in because she has a history of also, of sexual assault and stuff like that and she never turned any of the guys in so she didn’t understand why I was, in her words, ”Making a big deal out of nothing. “ I felt like I was letting her down because I was standing up for myself which was a dilemma in and of itself. Then when I finally was like, “I can’t live with myself and I can’t do this anymore. ” 

**McKenzie:** McKenzie shared that she had joined the Air Force because “when I was in high school, I started the Reserve Officers’ Training Corps (ROTC). And it was then that I knew that serving was for me. Before that, it’s been a thought that hadn’t been that driving force. And going through high school in ROTC and meeting the instructors that I did and the people that I did, I was able to find, I guess, the closest thing that I had to a family. Like that sense of belonging. And the more I delved into the military and how they live and what their job is and how what they do makes such a huge difference, I knew that that was where I was supposed to be. That was where I was supposed to be headed in doing. Before that, it was always I knew medical, but the military added to that medical interest in helping and making such a difference. It was what gave me purpose in life.” McKenzie shared that contextual features in contemplating suicide was when she was in a new location and unit and having a lot of stress in her unit. 

**Gummo:** Gummo shared that he served as a Marine for 12 years in special operations and with the Air Force as a pararescue man where he is in the “two or three percent” of the Air Force members whose daily job “is normally filled with the worst day of someone’s life”. One military experience of significance during his deployment to Afghanistan led to unanticipated hand-to-hand combat and deaths. Immediately after this significant event, through “serendipity”, Gummo was met coming off the helicopter when he returned to his overseas military location by three individuals from the “media” that were interested in learning about his unit’s experiences during deployment. One of the men was a tattoo artist that offered Gummo a tattoo. Gummo found the processes of receiving a tattoo as helpful by “riding into catharsis”. He shared there is limited time to process grief as there is little time now from being in battle and then chatting with his family back home in the United States. 

**John:** John shared that he is an Air Force pilot. “…For about six years…We were flying…in ground support missions.” John shared that he, in the past, suspected he may have benefited from mental health services; he was interested particularly in medication following his family connections to psychiatry, but policy dictates he would have been grounded and not allowed to fly. Rather than go to mental health and be taken off the mission and not fly, John shared he used alcohol to calm down. “I think I was lucky in that I was in a detox ward at the time. I had just come off of a four-day bender with alcohol. Over a four-day period I think I consumed probably five or six bottles of vodka. I was pretty much a wreck and out of sorts. I was in this detox ward. I was supposed to get out of there in four days. The counselors that were in the detox ward got ahold of my wife. She basically said we’re done, it’s time for a divorce, and some really other harsh language; but basically, you can sit in there and rot. At the time my daughter was getting up to 18 months, so I had a little baby at home. That was really tough for me”. 

**Charles:** Charles shared that he was serving the past 23 years in special operations as a Green Beret. Circumstances he shared of what contributed to the consideration of suicide was “survivor’s guilt, it goes back to that family, that person to your left and right, you’ve been with for so long, friend with for so long, and you’re wondering, you’re asking yourself why? Why, why him and not me. Having to relive it year after year”. 

**Hoppi:** “I’ve been a Green Beret and a special operator for many, many years… I come from the Northeast, coal mining country, where everybody worked hard, took pride in their work”. Hoppi shared that his consideration of suicide “Is not necessarily from shooting the enemy at all or seeing horrible things. A lot of it is stress compounded for so many years, because you’re trying to balance so many things, and you look at it as no fail. You cannot fail”. Hoppi also described the experience of being at peace in war and returning home as frustrating. “I was at peace over there because when something confronted me, I could kill it. Over here, you’ve got to deal with it. You can’t just grab somebody by the neck or bring in a B-1 bomber to end the problem. You have to listen to all of this madness, and it gets a little bit frustrating for when your normal is dealing with everything with extreme violence or force”. 

### 4.2. Effects of Saturation of Literature Pushing “Evidence Based” Structured Frameworks 

There is another dangerous idea that may be assumed in the language in the VA/DOD clinical practices that providers should use the same scripts to “Reduce current unwarranted practice variation and provide facilities with a structured framework to help improve patient outcomes (prevent suicide and other forms of suicidal self-directed violent behavior)” ([8], p. 3). The language in the quote locates suicide or bad outcomes as being caused by providers who don’t adhere to CBT or a step by step technician style manualized approach. This is an erroneous claim considering there are many theoretical practices, methods of diagnosis and assessment, and treatments that have historically produced successful outcomes too often disparaged and ignored by promoters of the dominant approach to mental health care [37,38,39]. Consequently, the saturation of CBT in the United States military training and research literature provides many privileges. CBT being economically advantaged in research is arguably the most widely studied form of psychotherapy [40] and produces more than 1000 research articles [41]. Mental health research is fueled by government funding, the government is historically the creator of the most research activities in the United States, spending billions annually [42]. Conversely, this saturation continues the controlling of the production of knowledge channels to continue the rhetoric of CBT as the few “evidence-based” practices and all other perspectives as located as illegitimate excluded from consideration on the grounds that they are not sufficiently scientific in the name of randomized controlled trials [37,38,43,44,45]. The suggested VA/DOD guidelines are created seemingly through this myth, thus other perspectives are made invisible and less privileged. 

Also embedded in the service members’ descriptions are the absences of a positive therapeutic relationship or meaningful therapeutic alliance with military mental health providers when confronted with unhelpful practices. The critical importance of the therapeutic alliance and relationship as primary mechanisms to improving outcomes in mental health services, regardless of the theoretical basis used by the mental health provider is well established [39,46,47,48,49,50,51,52,53]. The fact that members describe experiences related to policy that directly effects the ability for individuals and providers to engage in therapeutic relationships and to form meaningful alliances, one service member, Gummo, shared this was what led him to consider suicide is alarming. 

### 4.3. Stigma Created by DoD Policy

The descriptions of service members highlight military unique sociocultural features arising from DoD policy that contribute to the creation of stigma distinctive from that described and understood outside military contexts. Acosta et al. through their content analysis of DoD policies “identified policies that allow nonprofessionals to determine mental health fitness that support the use of mandated mental health screening for specific individuals or groups…these practices could put some service members at risk for stigma and discrimination” ([54], p. xix). They also state that “mental health screening and evaluation programs may be used inappropriately…” (p. xix). The descriptions of service members highlight military unique sociocultural features arising from DoD policy that contribute to the creation of stigma distinctive from that described and understood outside military contexts. Stigma attached to keeping people from seeking mental health services is not a new concept in the United States military. However, few military programs or policies currently target the nuances of the military institutional contexts [54], or other sociocultural factors described and illuminated by the participants’ experiences. This is most likely due to scarcity in the available literature describing stigma from the lens of intersectionality [28] or other perspectives to include the relational and contextual features arising from unique military circumstances that effect stigma over time. Of the available research literature, most researchers focus narrowly on the idea that service members are not presenting for mental health services due to misperceptions they have of mental health care, misperceptions of treatment, and misperceptions of career effects [29,55,56,57,58,59,60,61,62,63,64]. This locates the person as the creator of stigma in their head, thereby stigma is traditionally problem solved from this perspective. In the Department of Defense, this has led to funding programs and service wide messaging socializing that members who seek treatment early before it is a problem identifiable to others in their workplace have less career impact, and that seeking help is a sign of strength [5,6,29,31]. This fuels the idea that service members should engage in help seeking behaviors before it is a problem, which begs the question, why would people seek treatment if the problem is not a problem? 

Interestingly, there is a higher prevalence of stigma reported among service members in treatment, most likely due to (DODI) 6490.08 that mandates the lack of confidentiality in their workplaces through the process of seeking mental health services [54]. There seems to be the taken for granted idea in military research and messaging that service members’ misperceptions about treatment or misperceptions of career impact is what keeps people from seeking help, overlooking the policy nuances that occur when seeking mental health in the military also keeps people from being helped due to discriminatory social practices and policies as illuminated in the descriptions from the participants. Inadvertently, service members are trusting the messaging that seeking help comes with no strings attached without negative consequences; only that is not always the case. Acosta et al., in a study of mental health stigma in the United States military, warned of such dangers to service members after completing a content review of Department of Defense policies:

*“A large number of the policies we reviewed prohibited specific job opportunities or actions if a service member had a Mental Health Disorder (MHD) or sought mental health treatment. For many of these policies, the language is unclear, stating only that a service member is prohibited if he or she has a mental health issue…In 12% of policies, we identified language that was pejorative and characterized MHDs and treatment in a negative light.”*

([54], p. xxi).

Agreeing with Reavley and Jorm [65] that stigmatizing attitudes lead to negative feelings, stereotyping, and discriminatory behaviors; these mechanisms play out regarding service members in that they are treated differently as dictated by policy. Department of Defense policies unjustly blankets those seeking mental health services with sanctions [54], locating the service member as seemingly incompetent and untrustworthy, one who cannot manage successfully their lives and unable to manage their work duties. They are placed under circumstances of having to prove themselves capable, set up for failure as the policy mechanism demands them treated incapable, thereby they become incapable; their lives and personhood in essence being taken away. Regarding suicide prevention, this creates further suffering in those identified as suffering and wanting help, causing them to be discriminated against in the process. Policy inadvertently creates circumstances known to increase suicide risk to include isolation, loss of social support, loss of team membership, loss of purpose, and loss of hope and meaning [8,66,67].

## 5. Conclusions

### 5.1. Possible Ways Forward

Congruent with the aim of our study and the preferences of military policy leaders, to legitimize insider perspectives and knowledges to guide in transformations of policy, below are the recommendations in their own words, followed by ours. We also, considering the participant experiences of their stories being re-languaged, consequently regurgitated through other’s perspectives, with very real ramifications to their lives, we are intentionally leaving their recommendations as they uttered them. Future research and projects inspired by this work may take unlimited shapes and forms, and we hope to hear what projects are inspired from this manuscript.

**Aria:** Confidentiality needs to change. I think it’s a benefit of having a mental health clinic on base, it’s just better to have it off honestly. I have recommended seeking help outside of a base, rather than on a base, so that everybody is not able to stick their nose into your business.

**Charles:** I see all these different classes…Suicidal trainings, two or three times a year. Online training, two or three times a year. I don’t see that’s heavy here. That when I say heavy here, a person is standing up in front of the audience, telling that Soldier, Airmen, Seamen, Marine, look to the people on your left and right. Is there a person who you grew up with? These are the people you trained with, is it a people you fought with, these are the people you don’t let down. Not only that, but imagine. Put in your head who you love most and on this world. You love your mother the most, your father, your sister, your brother, your wife, your children, your grandchildren. Go ahead and pull that trigger. Imagine, the after effect is going to happen. Yes, sit there to think about. Contemplate that just for five minutes. Everything that’s going to happen, what are you initialized, what is for every action there’s a reaction. You pull that trigger, what are the reactions. Mentally what is it going to do, to that loved one. Mentally what is it going to do to your buddy, to your left and right. You might just be setting off something yourself, for the chain reaction of a lot more death that you don’t want to see. 

**Trish:** At my first duty station I tried really hard to get with the Top Three and the First Sergeant organizations and start a mentoring program due to the center that I was heading up because I really wanted to start pairing people with people who outranked them and had all this life experience in the military and expertise and knowledge that they had to offer to these young airmen who this was their first duty station and it was a terrible station and I had got orders and left in the middle of the process of getting all of that taken care of so I don’t actually know if it took off or not, but I did express the need for it because I knew my rank drastically changed once I got a mentor because I knew that no matter what happened, I could always call her, but I’m out of the military and I still call her.

**Hoppi:** Educate first-line supervisors to offer help interventions. The reason I say that is, it’s got to be the guy that’s with these individuals from morning to night. When you’re talking second, third, and fourth level up there, you will never be able to see it because the person is going to walk by you and say, ‘Good morning, Sergeant Major,’ and go about their day. It’s got to be the person that’s right beside him, that first-line supervisor that’s up in their rooms and with them every day. Yeah, they should be able to identify this. That self-medication is a huge indicator, when a guy starts drinking too much, it needs to be looked at because that’s normally one of the first things that starts happening. If you go to that first-line supervisor, in most cases, the soldier trusts that first-line supervisor with his wife and his life, he would be a little bit more open to taking recommendations or advice or just open dialogue with that first-line supervisor. When you get beyond that, now there’s a wall going up.

**McKenzie:** I shouldn’t be judged any differently as long as I can do what I’m supposed to be doing I shouldn’t be judged any differently. I know that there are some diagnoses that are unsuiting for a lot of things but there’s a lot of gray in the mental health focus as far as the military is concerned. They keep harping on the whole wingman concept and get help, be there for your people and I feel that it’s very fake in a lot of ways because from just my experience itself it’s been everything that they say it’s not. I would love to see that change because you can’t persist and say, “Hey we’re here to help you, we care about you, you’re a member of our family” if you will but then turn around and completely disregard everything you just said. That’s my two huge things is that as long as we’re still able to do what we’re supposed to be doing we shouldn’t be judged any differently. The fact that the whole air force philosophy of being honest and open and seeking help and being a wingman that needs to be followed. It’s not one of those things that you can just say because it sounds good. That’s why there’s a stigma is because people are concerned that they’re going to be just treated differently, that they’re going to risk their careers.

**John:** Advertise success stories. Advertise people that are on medication that are still in and it’s not damaging their career. I would try to advertise that. I think that would be helpful. Say if I read an article ten years ago that said, “Hey, this guy’s a pilot. He takes Lexapro. This guy’s a psychiatrist. He takes Prozac. This guy’s a brain surgeon. He’s on Zoloft. They’re all functioning and their careers are great. Oh, by the way, they feel great, too. They don’t have anxiety. They don’t have depression.” I think that would be huge. I still think the culture is very against it. It’s like, “Ew, you’re going to mental health? What’s wrong with you. Why do you have depression? What’s wrong with your life that your so depressed? Jeez, maybe I shouldn’t give this guy the job.” 

**Tirzah and Bernadette (researchers/authors of the manuscript):**

(1) Military policy organizations revamp their internal mechanisms to include speaking with service members in each military specific mission as a critical means to inform policy efforts. Each mission is different in the effects of certain policy and unit contexts as the participants’ experience highlighted.
(2) In suicide prevention efforts, include service members at the local level to participate in the national level conversation and vice versa. Annually or every few years, we suggest employing a three- or five-day solution finding event at local units, not specifically focused on suicide per se, but to better understand local occupational and/or other cultural stressors (pay, housing, deployment schedules, work and family balance, and other base specific nuances), thereby providing local solutions to these nuances. This information could be useful to collect for policy makers to also utilize to make decisions that are mission and unit specific.
(3) DoD add to their local and national measures individuals’ experiences of therapy, and services provided to inform ongoing policy and services transformations. This is to compliment already existing outcome measures captured by the military.
(4) Consider removing the command and supervisor directed mental health evaluation mandates in DoD policy.
(5) Consider updating the DoD policy related to confidentiality, particularly in the workplace requirements.
(6) Consider rethinking the narrow specificity of utilizing CBT therapies outlined in the VA/DoD Clinical Practice Guideline for Assessment and Management of Patients at Risk for Suicide. As the clinical guidelines indicate, there is no interventions more useful than others in stopping one from committing suicide [12]. In addition, all therapies work for some people and not others [37,38,39,43,44], the diversity in theoretical ideas and interventions could be celebrated in clinical practice guidelines.

### 5.2. Limitations of the Study

The seven service members who participated did so to work alongside military mental health policy makers. Readers should be mindful that the sociocultural factors and polices of the military are not static and are ever changing. In addition, in our criticality of policy and the services provided does not negate that some people find the policy and services as helpful. Our intent is not to have the reader assume we are advocating, through our situating ourselves in the critical and questioning stances, that we wish to categorically subscribe to naming things “good” or “bad”, “right” or “wrong”, or other words to the same effect. It is our position rather to raise up the unintended consequences of helpful items not helpful through the experiences of insiders to the process to inform policy. It is our moral and ethical obligation to keep an eye on the inadvertent ripples to the lives our policy and practices touch.

## Figures and Tables

**Table 1:** Themes and Subordinate Themes.

**Theme One: Lack of Confidentiality of Service Members in the Workplace**
Subordinate Theme A: Ripples of I’m tired of being told I can’t do something when I’m more than capable of doing it.
Subordinate Theme B: Units Being Super Supportive and/or Not
**Theme Two: Unit Members Surveillance and Command Directed Evaluations**
**Theme Three: Military Mental Health Services: Diagnosis and De-Contextualization of Experiences.**
Subordinate Theme A: Loss of Ability to Articulate Humanity to the Authority of Pathology Language.
Subordinate Theme B: I am your textbook and theory, study me
